# Seeking Synchrony Between Family Planning and Immunization: A Week-10 DMPA Start Option for Breastfeeding Mothers

**DOI:** 10.9745/GHSP-D-17-00063

**Published:** 2017-09-27

**Authors:** John Stanback

**Affiliations:** a FHI 360, Durham, NC, USA.

## Abstract

Many mothers initiate DMPA injectables at 6 weeks postpartum, at the time of their baby's first immunization visit. Offering an optional delayed DMPA start at the next (10-week) immunization visit has potential advantages including a reduced follow-up schedule with DMPA visits synchronized with other immunization visits, and, possibly, improved contraceptive and immunization outcomes.

The single most popular moment in Africa (and many other regions) to initiate family planning may well be the 6-week postpartum clinic visit, when mothers also bring infants to begin the crucial primary immunization series.^1^ A 6-week start does indeed work well for mothers accepting long-acting, reversible contraceptives (LARCs) such as implants and intrauterine devices (IUDs), because these methods have no negative impacts on breastfeeding and, once inserted, remain effective for years. However, although there are no medical restrictions on starting injectables at 6 weeks, the 6-week postpartum visit may *not* be the optimal timing for initiating injectables, the most popular method in sub-Saharan Africa, comprising nearly half of modern method use in the region.^2^

The 6-week postpartum visit may not be the optimal timing for initiating injectable contraceptives.

What is the potential downside of initiating depot-medroxyprogesterone acetate (DMPA) injectables at 6 weeks? Beyond the redundant use of contraceptives during lactational infertility^3^^,^^4^ is the problem of high discontinuation. In a review of Demographic and Health Survey (DHS) data from 19 countries, Ali et al.^5^ noted that more than 40% of new injectable clients discontinued within the first year of use. When such early discontinuation occurs among postpartum women—during the time infants are weaned and fertility is reestablished—the stakes are even higher, because these mothers need effective contraception for optimal birth spacing.

Although high injectable discontinuation has proven a particularly challenging problem to solve,^6^ several partial solutions present themselves for better protection during the first postpartum year. For example, more intensive counseling, particularly on the side effects that users can expect, has been shown to increase continuation rates among injectable users.^7^^,^^8^ Also, for the many women who use DMPA because more effective methods are not available, programs must continue to improve access to LARCs, particularly in rural areas where the poorest and most vulnerable live. Finally, we should do a better job promoting exclusive breastfeeding during the first 6 months postpartum and ensuring that those using the Lactational Amenorrhea Method (LAM) can smoothly transition to another effective method when desired.

There is another option that merits investigation. Fully or nearly fully breastfeeding mothers desiring the most popular injectable, DMPA, at 6 weeks could be offered the option of delaying their injection for 1 month, until the second visit of the scheduled 6-, 10-, and 14-week primary immunization series. Week-10 DMPA initiation has much to recommend it. For example, given existing discontinuation patterns, the delayed start time will translate into a delayed discontinuation time, meaning that mothers will have an extra month of contraceptive protection, more likely to fall at a time without redundant protection from lactational infertility. Furthermore, well-counseled clients who want to limit births or who want a highly effective spacing method will have an extra month to consider their family planning options and to discuss these options with their providers and partners. Upon return, they may be more likely to accept a more effective method and/or one with less chance of early discontinuation. Finally, when DMPA initiation is delayed until the second well-baby visit at 10 weeks, mothers benefit from better synchronization of clinic visits during the first year postpartum.

Fully or nearly fully breastfeeding mothers desiring DMPA at 6 weeks postpartum could be offered the option of delaying their injection until the second immunization visit at 10 weeks.

When DMPA initiation is delayed until the 10-week visit, mothers benefit from better synchronization of clinic visits during the first year postpartum.

## SYNCHRONIZED SERVICES

This last advantage, better synchrony, may be the most important, but the benefits of synchronized visits have never been promoted or evaluated, in spite of much recent attention focused on the benefits of integrating family planning with other health services such as immunization.^9^ Intuitively, initiating contraception during the 6-week visit makes good sense. Throughout the developing world, maternal and child health programs have made enormous investments to ensure that mothers come to clinics at this time for their infants' immunizations and growth monitoring, so it is no surprise that family planning programs have tried to “piggyback” onto this all-important visit.

**FIGURE 1:**
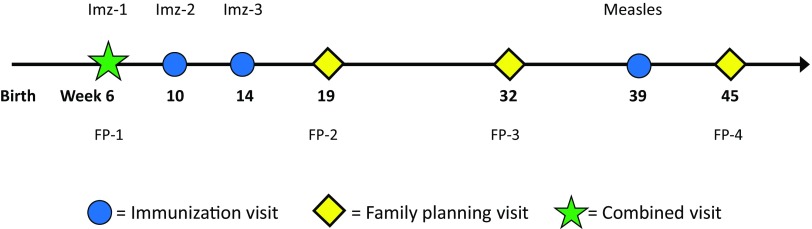
Standard Week-6 DMPA Initiation Schedule With 7 Immunization and DMPA Visits Through 11 Months Postpartum Abbreviations: DMPA, depot-medroxyprogesterone acetate; FP, family planning; Imz, immunization.

However, given the 3-month (13-week) cycle of DMPA, subsequent resupply visits after a week-6 initiation do not align well with scheduled immunization visits, resulting in a total of 6 scheduled family planning and immunization revisits in the following 10 months (Figure 1).

**FIGURE 2:**
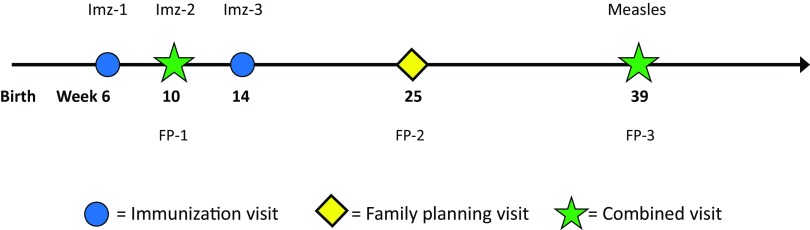
Optional Week-10 DMPA Initiation Schedule With 5 Immunization and DMPA Visits Through 11 Months Postpartum Abbreviations: DMPA, depot-medroxyprogesterone acetate; FP, family planning; Imz, immunization.

In contrast, if new mothers are counseled about family planning at week 6 and choose DMPA, they can be offered the option of a more “mother-friendly” schedule if they delay their first injection until their baby's second immunization visit at week 10. This voluntary alternative schedule (Figure 2), which takes modest advantage of the 1-month “grace period” for DMPA resupply, decreases the number of follow-up visits (after the initial 6-week visit) over the next 10 months by a full third—from 6 visits to 4 visits. (If the full 12 months postpartum period is considered, a final family planning visit would be due at week 52.) Furthermore, it is possible that women will better adhere to a reduced schedule of dual-purpose visits than to more numerous single-purpose visits. This could improve contraceptive continuation if scheduled immunization visits help overcome any hesitation to seek DMPA reinjection. Similarly, a synchronized schedule could boost immunization timeliness and coverage if the family planning appointment provides an extra cue to action for the 10-week booster or the often-neglected measles vaccination at 39 weeks. Finally, a scheduled mid-year family planning visit at around 6 months (25 weeks) (Figure 2) coincides with the baby's recommended first dose of vitamin A, and also presents a good opportunity for growth monitoring and counseling on complementary feeding at the World Health Organization's recommended timing for weaning.

Of course, there are several potential arguments *against* the 10-week start for DMPA. The first, that mothers may become pregnant between 6 and 10 weeks postpartum, should be negligible if providers are careful to offer this option only to fully (or nearly fully) breastfeeding mothers who plan to continue breastfeeding for several months. A more serious argument is that a mother who presents at 6 weeks postpartum might never return. This is certainly possible, but drop-out between the first and third visits in the primary immunization series is already low—an average of 6% in Gavi-supported countries^10^—and having 2 reasons to return at 10 weeks, instead of only 1 reason, could further reduce that drop-out rate.

It is much more likely that some mothers will be *late* for the 10-week visit. Indeed, a 2009 *Lancet* review of the timing of children's vaccinations in 45 low- and middle-income countries, while not explicitly addressing the 10-week visit, found that tardiness was common.^11^ However, such delays might actually be *reduced* by virtue of the dual-purpose nature of the visit—mothers not sufficiently motivated to be on time for their baby's vaccination boosters may be more motivated by their own contraceptive needs. Furthermore, even if mothers are late, fully or nearly fully breastfeeding should protect them from pregnancy for at least 6 months, if they experience no bleeding episodes.

## A CALL FOR RESEARCH

Compelling practical and theoretical arguments exist for giving breastfeeding mothers the option of delaying DMPA initiation from 6 weeks to 10 weeks postpartum. Research should be undertaken to test hypotheses related to the potential benefits of “synchronizing” the DMPA schedule with that for infant immunizations. Will new mothers accept a 1-month delay in initiating DMPA use? Will providers offer women this option, given the modest extra effort required? And, most importantly, will the benefits of synchronized, dual-purpose visits translate into better contraceptive continuation, immunization coverage, and other outcomes, compared with possible risks, such as unintended pregnancies among mothers who stop breastfeeding or fail to return?

Research should be undertaken to test the potential benefits of synchronizing the DMPA schedule with that for infant immunizations.

Synchronized scheduling for new mothers, with fewer, more integrated revisits, not only reflects the tenets of integrated services and patient-centered, mother-friendly care, but could also improve important outcomes in vulnerable populations.

## Notes

**Peer Reviewed**
